# Differences between Well-Differentiated Neuroendocrine Tumors and Ductal Adenocarcinomas of the Pancreas Assessed by Multi-Omics Profiling

**DOI:** 10.3390/ijms21124470

**Published:** 2020-06-23

**Authors:** Teresa Starzyńska, Jakub Karczmarski, Agnieszka Paziewska, Maria Kulecka, Katarzyna Kuśnierz, Natalia Żeber-Lubecka, Filip Ambrożkiewicz, Michał Mikula, Beata Kos-Kudła, Jerzy Ostrowski

**Affiliations:** 1 Department of Gastroenterology, Pomeranian Medical University in Szczecin, 70-204 Szczecin, Poland; testa@pum.edu.pl; 2 Department of Genetics, Maria Sklodowska-Curie National Research Institute of Oncology, 02-781 Warsaw, Poland; pocztakuby@gmail.com (J.K.); agapaziewska@poczta.onet.pl (A.P.); mkulecka@cmkp.edu.pl (M.K.); filip.ambrozkiewicz@coi.pl (F.A.); michal.mikula@coi.pl (M.M.); 3 Department of Gastroenterology, Hepatology and Clinical Oncology, Centre of Postgraduate Medical Education, 01-813 Warsaw, Poland; natalia.zeber@o2.pl; 4 Department of Gastrointestinal Surgery, Medical University of Silesia, 40-514 Katowice, Poland; kkusnierzchir@gmail.com; 5 Department of Endocrinology and Neuroendocrine Tumors, ENETS Center of Excelence, Department of Pathophysiology and Endocrinology, Medical University of Silesia, 40-514 Katowice, Poland; bkoskudla@sum.edu.pl

**Keywords:** neuroendocrine tumors, adenocarcinoma, NGS, mRNA, miRNA, somatic mutation

## Abstract

Most pancreatic neuroendocrine tumors (PNETs) are indolent, while pancreatic ductal adenocarcinomas (PDACs) are particularly aggressive. To elucidate the basis for this difference and to establish the biomarkers, by using the deep sequencing, we analyzed somatic variants across coding regions of 409 cancer genes and measured mRNA/miRNA expression in nine PNETs, eight PDACs, and four intestinal neuroendocrine tumors (INETs). There were 153 unique somatic variants considered pathogenic or likely pathogenic, found in 50, 57, and 24 genes in PDACs, PNETs, and INETs, respectively. Ten and 11 genes contained a pathogenic mutation in at least one sample of all tumor types and in PDACs and PNETs, respectively, while 28, 34, and 11 genes were found to be mutated exclusively in PDACs, PNETs, and INETs, respectively. The mRNA and miRNA transcriptomes of PDACs and NETs were distinct: from 54 to 1659 differentially expressed mRNAs and from 117 to 250 differentially expressed miRNAs exhibited high discrimination ability and resulted in models with an area under the receiver operating characteristics curve (AUC-ROC) >0.9 for both miRNA and mRNA. Given the miRNAs high stability, we proposed exploring that class of RNA as new pancreatic tumor biomarkers.

## 1. Introduction

Pancreatic neuroendocrine tumors (PNETs) arise from the endocrine portion of the pancreas, and pancreatic ductal adenocarcinomas (PDACs) originate from epithelial cells. Although most are indolent, PNETs are unpredictable, ranging from nearly benign to aggressive, metastasizing neoplasms. Larger tumor size, higher grade, and liver metastases indicate a less favorable prognosis [1,2]. While the majority of PNETs are moderately malignant and slow-growing, PDACs, which account for more than 90% of pancreatic malignancies, are particularly aggressive, with rapidly infiltrative growth [3,4]. Nonfunctional PNETs and PDACs are characterized by a relatively long asymptomatic course, and, therefore, diagnosis of both types of pancreatic neoplasia is often made in advanced tumor stages. PDAC remains one of the deadliest cancer types. Trends for PDAC incidence and mortality significantly varies in the world, with the highest incidence and mortality rates in developed countries and is linked to a range of risk factors, such as smoking, obesity, genetics, diabetes, “western diet”, and physical inactivity [5,6]. While PDACs exhibit the lowest relative 5-year survival rate among all common cancer types, reaching to only about 9% [7,8], PNET patients even at the metastatic stages of the disease maintain relatively good general condition without cachexia symptoms and 5-year survival of 42% [9]. These major differences in the biological behaviors of advanced PNETs and PDACs are not fully understood.

The histological diagnosis of neuroendocrine tumors is confirmed by immunohistochemical analysis of chromogranin A and synaptophysin expression and the Ki-67/MIB1 proliferation index [1,3]. However, the histological differentiation grade (G) is the most important clinical characteristic [10]. Accordingly, the WHO 2017 classification system divides neuroendocrine tumors (NETs) into two basic groups: well-differentiated neoplasms (NET G1 and NET G2, with a proliferation index below 20%) and highly heterogeneous, poorly differentiated neoplasms (NET G3 with a Ki-67 proliferation index above 20%), referred to as neuroendocrine carcinomas (NECs) [11]. PDAC consists of atypical tubular glands, resembling medium-sized or smaller pancreatic ducts with heterogeneous growth patterns among and within tumors [12]. PDAC can include non-tubular components, such as a clear-cell, cribriform, or gyriform component, which may have an impact on patient survival [13], and the irregular tumor glands of PDAC are often embedded in a prominent desmoplastic stroma [14]. Histopathological grading of PDAC (according to defined WHO criteria) includes the presence of tubular structures vs. solid growth, the presence of mucin, nuclear polymorphism, and the number of mitoses [11].

Complex genetic and epigenetic changes are involved in the initiation and progression of both PDAC and NETs; the malignant behavior of neoplastic cells is driven by somatic mutations in oncogenes and tumor suppressor genes that affect cellular pathways, including cell growth regulation, cell interaction with the extracellular environment, and DNA repair systems [15]. The most frequently altered driver genes in PDACs are *KRAS, TP53, CDKN2A*, and *SMAD4*, while several other genes are mutated in only a small fraction of tumors; of these, only some can be drivers of pancreatic tumorigenesis [16,17,18,19]. The whole-genome sequencing (WGS) of non-familial PNETs has demonstrated somatic mutations in *MEN-1*, encoding menin, a component of a histone methyltransferase complex; in genes encoding either of the two subunits of a transcription/chromatin remodeling complex consisting of DAXX (death-domain associated protein) and ATRX (alpha thalassemia/mental retardation syndrome X-linked); in genes encoding components of the mTOR (mammalian target of rapamycin) signaling pathway [20]. Another whole-exome sequencing (WES) study has uncovered somatic point mutations and gene fusions in genes involved in four main pathways: chromatin remodeling, DNA damage repair, activation of mTOR signaling, and telomere maintenance [21]. The genomic and transcriptomic profiles of liver metastases from PNETs have identified biallelic loss of *MEN1* and *DAXX* and focal amplification of MYCN concomitant with loss of *APC* and *TP53*, suggesting that different cellular pathways may contribute to PNET progression [22].

To elucidate the basis of the distinct clinical courses of PNETs and PDACs, we compared the molecular signatures of mostly well-differentiated NETs and PDACs using multi-omics profiling.

## 2. Results

### 2.1. Mutational Status

To compare potential driver mutations in neuroendocrine tumors and ductal adenocarcinomas of the pancreas, targeted sequencing of 409 genes was performed in 13 NETs (nine PNETs and four INETs) and 8 PDACs; detailed characteristics of the patients are shown in Table 1.

The median mapped read count was 20,820,942. The median percentage of bases with greater than 100× coverage was 98.41%. Each read was assigned to its respective amplicon to perform mutation calling and estimate the mutant allele frequency.

**Figure 1:**
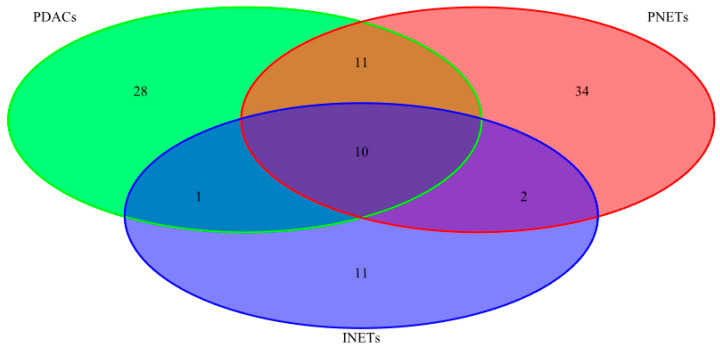
Venn diagram, depicting unique and shared gene numbers carrying deleterious variants across tested tissues. Parallel sequencing of 13 neuroendocrine tumors and 8 ductal adenocarcinomas of the pancreas identified 1288 unique variants across 234 genes, of which 50, 57, and 24 genes carried variants likely to be deleterious in pancreatic ductal adenocarcinomas (PDACs), pancreatic neuroendocrine tumors (PNETs), and intestinal neuroendocrine tumors (INETs), respectively.

After quality filtering, we identified 1288 unique variants across 234 genes, ranging from 106 to 206 variants for each tumor sample. Among them, 153 were considered pathogenic or likely pathogenic according to the VAST or CHASM algorithm or had a high impact on protein coding (stop-gained, frameshift, etc.) (Table S1). Of these, variants likely to be deleterious were found in 50, 57, and 24 genes in PDACs, PNETs, and INETs, respectively. Ten genes and 11 genes contained a pathogenic or likely pathogenic mutation in at least in one sample of all tumor types and in PDACs and PNETs, respectively, while 28 genes, 34 genes, and 11 genes were found to be mutated only in PDACs, PNETs, and INETs, respectively (Figure 1, listed in Table 2).

**Figure 2:**
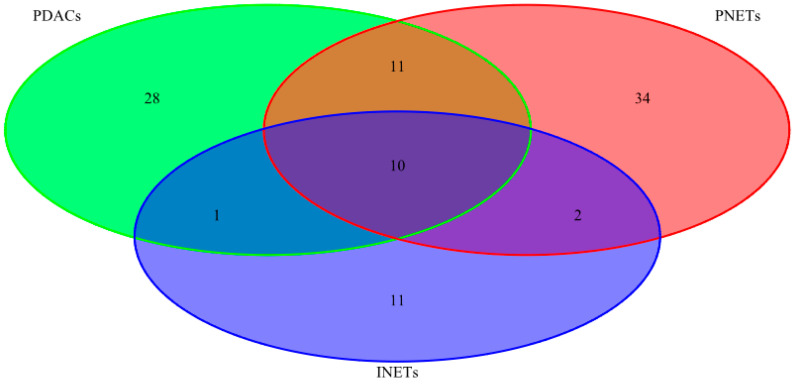
Venn diagram with variant numbers found in at least 40% of samples in all three tumor types selected by the parallel sequencing of 13 neuroendocrine tumors (nine PNETs and four INETs) and 8 ductal adenocarcinomas of the pancreas.

The seven most frequently mutated genes (*HNF1A, CREBBP, RNF213, KRAS, CASC5, APC,* and *TP53*) were found in 50% or more of PDAC samples. Two genes (*CASC5, APC*) and six genes (*HNF1A, CREBBP, KAT6B, MLL2, MLL3,* and *JAK3*) were found to be mutated in 44% and 33% of PNET samples, respectively. Eleven genes (*STK11, CASC5, CREBBP, RNF213, PAX7, NFKB2, FLT4, NOTCH1, FLT3, ZNF521,* and *EP300*) were found to be mutated in two of four INET samples. Of these, the most frequently mutated genes were *CREBBP* and *CASC*5 in all three tumor types, *HNF1A* and *APC* in both PDACs and PNETs, while two, four, and eight genes were mutated only in PDACs, PNETS, and INETs, respectively (Figure 2, Table 3).

Of genes previously reported as commonly mutated in PNETs, only *PDGFRA* and *MTOR* were found to be mutated in a few tumor samples, and other genes, including *ATM, ATRX, TSC2, DAXX, VHL*, and *MEN1*, were mutated only in individual samples.

### 2.2. Gene Expression Analyses

**Figure 3:**
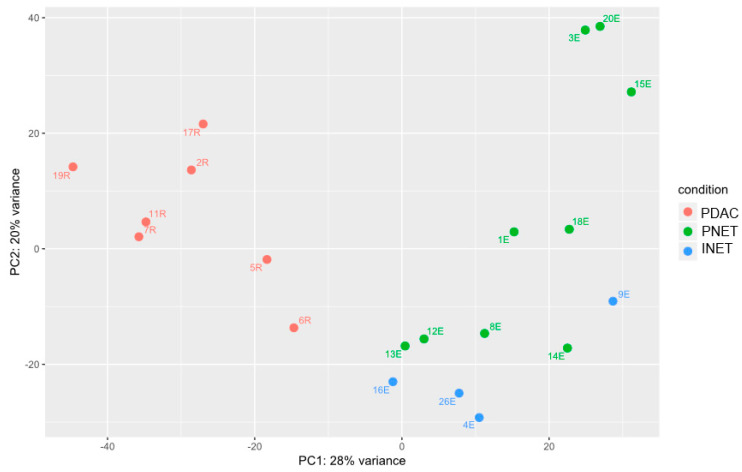
Deep sequencing of the mRNA transcriptomes identified 2082, 2621, and 107 mRNAs, whose expression significantly differentiated between PDACs and PNETs, PDACs and INETS, and PNETs and INETs, respectively, as demonstrated by principal component analysis (PCA) using the DESeq2 function “plotPCA” with the default parameters.

We analyzed gene expression in tissue samples by deep sequencing of the mRNA and miRNA transcriptomes. Differentially expressed genes were identified using Wald’s test, and principal component analysis (PCA) of these genes revealed visible separation of PDACS from NETs and some, but not so clear, second principal component separation of PNETs from INETs (Figure 3).

The pairwise comparison revealed that 2082 (1372 downregulated and 710 upregulated), 2621 (1966 downregulated and 655 upregulated), and only 107 (86 downregulated and 21 upregulated) mRNAs differentiated PDACs and PNETs, PDACs and INETS, and PNETs and INETs, respectively (Table S2). There were 1235 common to PNETs and INETs compared with PDACs.

There were 798, 1659, and 54 differentially expressed mRNAs that exhibited a high ability to discriminate between PDACs and PNETs, between PDACs and INETs, and between PNETS and INETs, respectively, with an area under the receiver operating characteristics curve (AUC − ROC) > 0.9 (Table S2); of these, 92, 494, and 16 discriminated between tumor samples in the respective pair comparisons with an AUC − ROC = 1.0.

RNAseq data sets were functionally analyzed by annotation to the Reactome signaling pathway database. Several Reactome pathways were found to be discriminative between PDACs and PNETs. The genes that were significantly downregulated in PNETs compared with PDACs could be classified into 53 functional groups (Table S3). The first 10 significantly overrepresented Reactome pathways included genes from five groups: interleukin-10 signaling, immune system, extracellular matrix organization, degradation of the extracellular matrix, and syndecan interactions (Table S4). Upregulated transcripts could be described with 25 functional groups. Here, the first 10 significantly overrepresented Reactome pathways included genes from five groups: neuronal system; GABA synthesis, release, reuptake and degradation; neurotransmitter receptors and postsynaptic signal transmission; GABA receptor activation; regulation of gene expression in beta cells (Tables S5 and S6).

**Figure 4:**
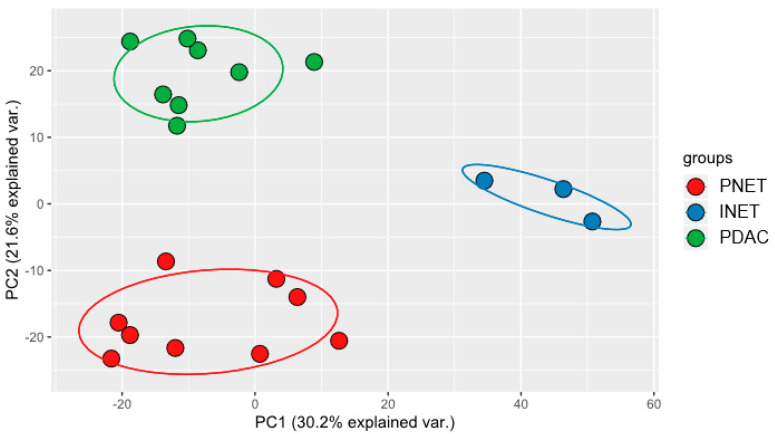
Deep sequencing of the miRNA transcriptomes identified 258, 297, and 129 mRNAs, whose expression significantly differentiated between PDACs and PNETs, PDACs and INETS, and PNETs and INETs, respectively, as demonstrated by principal component analysis (PCA) using the standard R programming language function “prcomp.”.

On average, 1,161,543 reads mapped to miRBase were obtained per library, representing 41% of total reads. Altogether, 1532 mature miRNAs were detected, of which 452 generated 10 reads on average. The pair-wise comparisons identified in total 258, 297, and 129 significantly dysregulated miRNAs (adjusted *p*-values < 0.05) between PDACs and PNETs, PDACs and INETS, and PNETs and INETs, respectively (Table S7). There were 157 miRNAs common to PNETs and INETs when compared with PDACs. PCA analysis of the differentially expressed miRNAs revealed a clear separation between PDACS, PNETs, and INETs (Figure 4).

There were 126, 250, and 117 differentially expressed miRNAs that were highly discriminative between PDACs and PNETs, between PDACs and INETs, and between PNETS and INETs, respectively, with AUC-ROC 0.9 (Table S8). Of these, 26, 176, and 89 miRNAs discriminated between tumor samples in the respective pair comparisons with an AUC-ROC = 1.0.

### 2.3. Integrated Analysis

**Figure 5:**
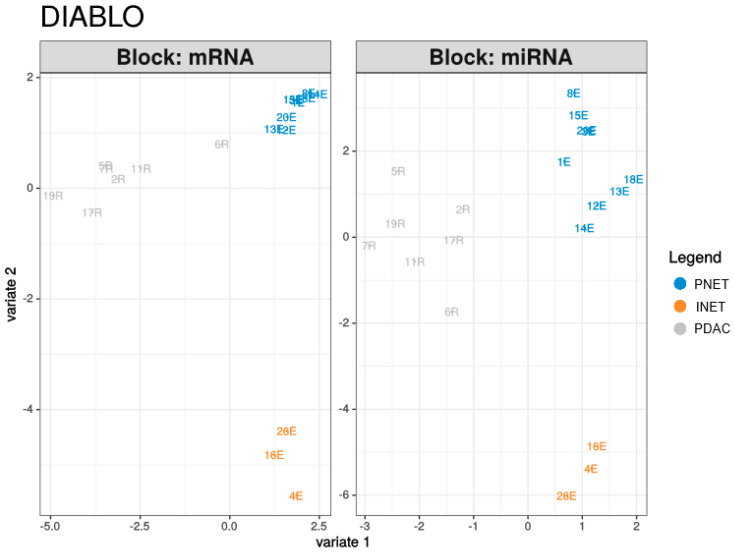
Scatter plot of individual samples from the DIABLO PLS-DA analysis results.

**Figure 6:**
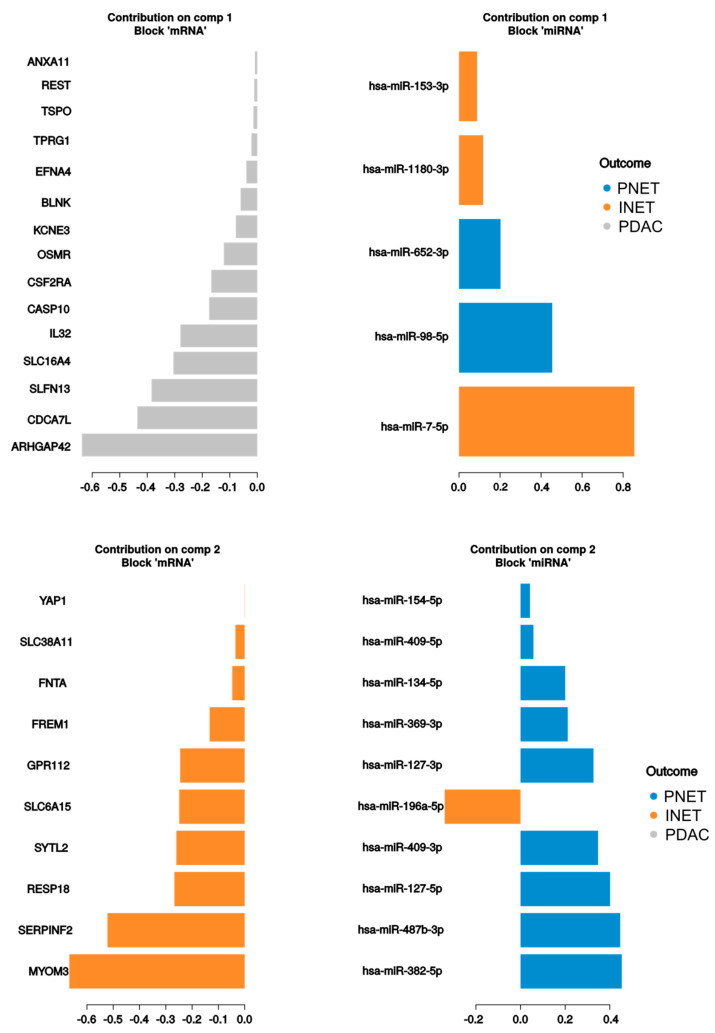
Main contributors to the first two components of the DIABLO PLS-DA model for miRNA and mRNA.

The PLS-DA analysis resulted in models with high discriminatory powers (AUC = 1) for both miRNA and mRNA in the second component (Table 4, Figure 5). There were in total of 25 contributors to the first and second components from mRNA and 15 from miRNAs (Figure 6). mRNAs differentiated mostly PDACs and PNETs from other groups, while miRNAs contributed mostly to PNET and INET differentiation. In addition, 220 correlations with absolute coefficient values above 0.4 were found between contributors to the first three components, of which 175 were negative correlations. This might suggest negative regulation by miRNAs (Table S8, Figure 1).

## 3. Discussion

PNETs are relatively uncommon neoplasms, constituting 1–5% of pancreatic tumors [9,23,24]. Two major categories of PNETs are well-differentiated NETs and poorly-differentiated neuroendocrine carcinomas (NECs) [25]. In contrast to aggressive PDACs with short survival, PNETs represent neoplasms of a relative indolent clinical course characterized by slow tumor growth [25]. Although a possible common origin between PDACs and PNETs may exist [26,27,28], deep differences between well-differentiated NETs and endocrine carcinomas, as well as adenocarcinomas, have been reported with regard to mutations and gene expression [25,29].

Our massive parallel sequencing of 13 NETs, 11 of which were well-differentiated, and 8 PDACs identified 1288 unique variants across 234 genes. Of these, proliferating driver mutations were present in a fraction of genes, and variants that were considered pathogenic or likely pathogenic were found in 57, 24, and 50 genes in PNETs, INETs, and PDACs, respectively. The most mutated genes in the PDAC samples were *HNF1A, CREBBP, RNF213, KRAS, CASC5, APC*, and *TP53*; in the PNET samples, *CASC5, APC, HNF1A, CREBBP, KAT6B, MLL2, MLL3*, and *JAK3*; in the INET samples, *STK11, CASC5, CREBBP, RNF213, PAX7, NFKB2, FLT4, NOTCH1, FLT3, ZNF521*, and *EP300*. While mutations in *HNF1A, CREBB, RNF213, CASC5*, and *APC* were shared by NETs and PDACs, mutated *KRAS, TP53*, and *SMAD4* were found only in PDACs.

As reported previously, recurrent mutations of *MEN1* and *VHL* are identified in distinct NET hereditary syndromes, and those involving *DAXX, ATRX* (found in a mutually-exclusive manner in pNETs) [30], and mTOR pathway genes (*PTEN, TSC2, NF1, PIK3CA*) are commonly found in sporadic PNETs [21,31,32]. In turn, in most sporadic and familial PDACs, four key driver mutations in *KRAS, TP53, SMAD4*, and *CDKN2A*, and mutations in other oncogenes (c-myc, PAK4, HER2), tumor suppressor genes (*PTEN, BRCA2, PALB2*, and *PRSS1*), and DNA mismatch repair genes (*MLH1, MSH2, MSH6*, and *PMS2*) have been found [9]. As described by the International Cancer Genome Consortium (ICGC), whole-genome and deep-exome sequencing analyses of 456 PDACs [33] have revealed repeated mutations in cancer genes at the following frequencies: *KRAS*, 89.8%; *TP53*, 66.1%; *SMAD4*, 22.5%; *CDKN2A*, 18.5%; *ARID1A*, 7.6%; *LRP1B*, 5.7%; *RNF43*, 5.5%; *KMT2C*, 5.5%; *KMT2D*, 5%. A study using a comprehensive cancer gene panel has found the most frequent somatic mutations in PDACs in *KRAS, PIK3CD, TAF1L, MTOR*, and *TP53* [4]. Thus, while activating mutations of *KRAS* and inactivating mutations of *RB1* and *TP53* are typically absent in well-differentiated PNETs, they are commonly seen in both PDACs and pancreatic neuroendocrine carcinomas [29,33,34,35,36,37].

Whole-genome sequencing of sporadic PNETs, mostly of G1–G2 grade, revealed that *MEN1, DAXX*, and *ATRX* were mutated in 36.7%, 22.4%, and 10.2% of tumors, respectively, and that *PTEN, SETD2, ATM, MTOR, NUMA1, TP53*, and *KMT2C* were mutated at a frequency between 7.1 and 3.1% [21]. Of these genes, *MEN1* and *DAXX* were found to be mutated in one-fifth of our PNET samples; other genes, including *ATRX, PGFRA, MTOR, ATM, TSC2*, and *VHL*, were mutated in individual samples. In summary, we demonstrated that the mutational burden of NETs did not differ from that of PDACs, and while some of the mutated driver genes were shared between all three tumor types, others differentiated one tumor type from the other two.

In contrast to genetic analysis, deep sequencing of the mRNA and miRNA transcriptomes identified much more visible separation between PDACS and NETs at the molecular level. Of 20,520 mRNAs and 1532 mature miRNAs identified by RNAseq, 2082, 2621, and 107 mRNAs and 258, 297, and 129 significantly dysregulated miRNAs differentiated PDACs and PNETs, PDACs and INETS, and PNETs and INETs, respectively. In addition, 220 correlations were found, mostly negative, between relative levels of mRNAs and miRNAs for variables that contributed to three components in DIABLO analysis.

The rarity of PNETs has limited the number of large-scale transcriptomic studies, in contrast to numerous studies previously conducted in pancreatic cancer. Bioinformatics analysis of the two data sets from ICGC and two from the NCBI GEO database has identified the five common differentially expressed genes in PNETs (ABCC8, PCSK2, IL13RA2, KLKB1, and PART1) related to their development and prognosis [38]. A microarray-based study, performed on 72 primary PNETs, 7 matched metastases, and 10 normal pancreatic samples, has found that TSC2 and PTEN—two important inhibitors of the Akt-mTOR pathway—are downregulated in PNETs, and FGF13 is upregulated in metastatic compared to nonmetastatic primary tumors [39]. Both mouse and human well-differentiated islet/insulinoma tumors express genes characteristic of mature islet cells, while poorly differentiated tumors associated with liver metastases express genes known to regulate early pancreatic development [40]. Another microarray-based analysis of gene expression patterns in PNETs has shown that upregulated, differentially expressed genes are related to several pathways, including type 2 diabetes mellitus, Ca^2+^ signaling, long-term potentiation, and long-term depression, and that downregulated genes are enriched in pancreatic secretion, protein digestion and absorption, and other metabolic pathways. Interferon-stimulated gene protein 15, somatostatin, and synaptosomal-associated protein 25 kDa are identified as hub proteins [41].

When our mRNAseq data sets were annotated according to Reactome signaling, downregulated transcripts in PNETs, when compared with PDACs, were classified into 53 functional groups, including interleukin-10 signaling, immune system, extracellular matrix organization, degradation of the extracellular matrix, and syndecan interactions. Upregulated transcripts fell into 25 groups, including the neuronal system; GABA synthesis, release, reuptake, and degradation; neurotransmitter receptors and postsynaptic signal transmission; GABA receptor activation; regulation of gene expression in beta cells (Tables S4 and S6). These findings proved differences in the biology of well-differentiated neuroendocrine tumors and pancreatic adenocarcinomas, which might relate to different tumorigenesis pathways.

In fact, NETs originate from the diffuse neuroendocrine system, and functional NETs can secrete different peptides and amines categorized as nonspecific markers (e.g., chromogranin A, neuron-specific enolase, and pancreatic polypeptide) and specific markers (e.g., serotonin, gastrin, insulin, glucagon, somatostatin, and vasoactive intestinal peptide). Our functional annotations of differentially expressed genes confirmed significant biological differences between PNETs and PDACs.

Each of the approximately 1800 human miRNAs may have regulatory roles in multiple cell signaling pathways, and some mediate and/or their expression correlates with oncogenesis. Oncogenic miRNAs are linked to several pro-tumorigenic processes, such as apoptosis resistance, cell proliferation, chemoresistance, and EMT, depending on the function of their respective target mRNAs [42]. miRNAs can function as both oncogenes and tumor suppressor genes [43,44,45,46,47,48]. The analysis of miRNAs from a total of 76 gastroenteropancreatic NETs and 31 lymph node and 14 solid organ metastases has uncovered eight upregulated (let-7e, miR-126, miR-127, miR-30a-3p, miR-409-3p, miR-539, and miR-652, miR-95) and five downregulated (miR-155, miR-193b, miR-28-3p, miR-642, and miR-886-5p) miRNAs in PNETs. Of these, miR-126 and miR-155 are also reported as dysregulated in pancreatic adenocarcinomas [49,50]. Metastases are associated with elevated levels of miR-30a-5p, miR-210, miR-339-3p, miR-345, and miR-660; miR-150, miR-21, and miR-660 have shown a strong correlation with proliferation index and metastatic disease, in general, with each anatomic location (primary or metastatic) having one or more site-specific microRNAs [51]. Eight of these miRNAs (or miRNAs belonging to those families) were also differentially expressed in our study when PNETs were compared with PDACs, namely, hsa-miR-127-3p, hsa-miR-409-3p, hsa-miR-539, hsa-miR-652, hsa-miR-95, hsa-miR-155-5p, hsa-miR-28-3p, and hsa-miR-642. All those miRNAs exhibited high discriminatory power (AUC above 0.85). MiR-127-3p has been shown to exhibit a tumor suppressor function in osteosarcoma [52], giant cell tumors of bone [53], prostate cancer bone metastasis [54], epithelial ovarian cancer [55], and glioblastoma [56]. miR-409-3p inhibits cell proliferation and invasion of osteosarcoma [57], tongue squamous cell carcinoma [58], breast cancer [59], and prostate cancer [60]. miR-539 might be a tumor suppressor in hepatocellular carcinoma (HCC) [61], nasopharyngeal carcinoma [62], pancreatic [63], and colorectal cancer [64]. miR-652 is a critical regulator of proliferation and metastasis in endometrial cancer [65] and functions as an oncogene in gastric cancer [66], melanoma [67], and non-small cell lung cancer (NSCLC) [68]. Overexpression of miR-155-5p characterizes most solid and hematological malignancies [69,70], and dysregulation of miR-28 has been demonstrated in various types of human malignancies [71,72,73]. miR-95 has been shown to act as an oncogene in HCC [74] and NSCLC [75] and as a potential tumor suppressor in osteosarcoma [76]. In addition, the expression of miR-642 has correlated with Ki67 score in PNET [77]. In addition, hsa-miR-30a-3p and hsa-miR-193b have differentiated between PDACs and INETs. Other studies have revealed increased expression of miR-21, miR-103, miR-107, miR-146a, miR-142-3p, miR-142-5p, and miR-193b and lower expression of let-7 miR and miR-155 in PNETs [78,79,80,81,82]. However, in our study, the expression of hsa-miR-21, hsa-miR-146a-5p, and hsa-miR-142-3p was significantly lower in PNETs compared with PDACs. PNETs with higher expression of miR-196a have a higher pathological stage, mitotic rate, and Ki-67 index [83]. miR-19, miR-129-5p, miR-10b, and miR-200 have been generally implicated in cancer progression [84,85].

miRNAs profiling may have diagnostic and prognostic potential, correlating with cancer clinical outcomes [51]. Our own research, performed on three independent cohorts of patients, allowed the identification of diagnostic miRNAs in prostate cancer, of which miR-32-5p might discriminate benign prostate tissue from noncancerous areas within cancer-bearing prostates [86,87]. However, before this new biomarker would be ready for clinical use, further studies on its clinical benefits (including comparative- and cost-effectiveness) are required. When looking for a PNET diagnostic biomarker, similar studies are especially challenging due to the rarity of PNETs, and the recruitment of a sufficient number of patients would be only possible within a multi-center consortium.

## 4. Materials and Methods

### 4.1. Patients

A total of 21 patients were enrolled in this study from January 2018 to December 2018, including 9 with PNETs, 8 with PDACs, and 4 patients with intestinal neuroendocrine tumors (INETs); of the latter, one tissue sample was obtained from liver metastasis. All cases had adequate clinical and pathologic information. The patient’s sex, age, tumor site, size, stage, histology, grade, type of surgery performed, additional treatment, and follow-up data were recorded. Tumor grade and stage were evaluated according to the criteria of the 8th edition of the AJCC staging manual (2017) (www.cancerstaging.org ajcc@facs.org). All subjects provided written informed consent prior to participation, and the study complied with the Declaration of Helsinki. The study was approved by the ethics committee (decisions: Bioethics Committee of the Pomeranian Medical University kB-0012/32/14, dated 17 March 2014 and Bioethics Committee of Medical University of Silesia KNW/0022/KB1/102/II/17/19, dated 12 November 2019). Written informed consent was obtained from all participants.

*PNET patients.* The median age of PNET patients (8 women and 1 man) was 45.4 years (range 24–70). The median tumor size was 2.9 cm (range 1.3–3.5); five tumors were located in the pancreatic tail. Two tumors were G1, six were G2, and one was G3, respectively. Six tumors were at stage II, one at stage III, and one at stage IV. In all, but one, patients, distal pancreatic resection or pancreaticoduodenectomy was performed.

*PDAC patients.* The median age of PDAC patients (6 women and 2 men) was 60.3 years (range 38–72). The median tumor size was 2.7 cm (range 1.7–3.3); six tumors were located in the pancreatic head. All tumors were classified as ductal adenocarcinoma. Five tumors were G2, and six tumors were at stage III and 2 at stage IV. In five patients, potentially curative surgery was performed, and in three others, the only laparotomy.

*INET patients.* The median age of the INET patients (one woman and three men) was 64 years (range 59–67). The median tumor size was 2.6 cm (range 2–3). Three primary tumors were G1, and one liver metastatic tumor was G3. There was one tumor at stage II, one at stage III, and one at stage IV.

All tissue samples for molecular analysis were unfixed postoperative specimens, and all, but one, were primary tumors. The tissue was frozen in liquid nitrogen and stored until use at –70 °C.

### 4.2. Nucleic Acid Isolation

Total nucleic acids were isolated from tumor tissue specimens using the ALLPrep DNA/RNA Micro Kit (Qiagen, Hilden, Germany), following the manufacturer’s protocol. DNA sample concentrations were measured using a NanoDrop spectrophotometer, following the manufacturer’s instructions, and the DNA was stored at –20 °C. The purity and quantity of total RNA were measured using a Qubit Fluorometer and assessed using an Agilent 2100 Bioanalyzer with an Agilent RNA 6000 Nano Kit (Agilent, Santa Clara, CA, United States). RNA samples were stored at –70 °C.

### 4.3. Profiling of DNA Variants

Ion AmpliSeq Comprehensive Cancer Panel (ThermoFisher Scientific, Waltham, MA, United States) libraries were prepared from DNA samples for the analysis of the coding regions of 409 oncogenes and tumor suppressor genes by sequencing on an Ion Proton sequencer with the Ion PI Hi-Q Chef Kit, loading four samples onto an Ion PI Chip, as described previously [54].

### 4.4. Data Analysis and Variant Calling

Raw reads were processed using the Torrent Suite analysis pipeline version 5.10 and mapped to human genome assembly hg19 using TMAP [88]. Variant calls were made using Ion Reporter Software [89] (version 5.10), using the default parameters for somatic variants. Called variants were filtered using bcftools (version 1.3) and parameters provided in Table S9. Variants were annotated, and their functional consequences were predicted using Cravat [90] version 4.3. Only rare (i.e., with minor allele frequency less than 2%) and non-synonymous variants were analyzed further. From these variants, potential driver mutations were chosen based on one of the following criteria: (i) the CHASM *p*-value after FDR correction was smaller or equal to 0.1 for a single variant, (ii) the variant was a non-synonymous variant in a known driver gene, or (iii) the variant was pathogenic according to the VEST algorithm.

### 4.5. Profiling of Transcriptome Panels

mRNA and miRNA cDNA libraries were prepared with the Ion AmpliSeq Transcriptome Human Gene Expression Panel and Ion Total RNA-Seq Kit v2 for Small RNA Libraries, respectively, and an Ion Xpress™ RNA-Seq Barcode Kit (ThermoFisher Scientific, Waltham, MA, United States), according to the manufacturer’s protocol. The amplified libraries of ligation products (94–114 bp) were assessed on the Bioanalyzer 2100 using a High Sensitivity DNA Kit (Agilent, Santa Clara, CA, United States). Eight mRNA barcoded library templates at a concentration of 50 pM and up to 16 miRNA libraries at a concentration of 40 pM were loaded onto Ion PI chips and sequenced on an Ion Proton Sequencer (ThermoFisher Scientific, Waltham, MA, United States) with the Ion PI Hi-Q Chef Kit, according to the manufacturer’s instructions.

### 4.6. mRNA Data Analysis

Raw reads were processed using the Torrent Suite analysis pipeline and mapped to the AmpliSeqTranscriptome version of the human genome assembly hg19 by use of TMAP. Reads corresponding to each gene were counted using htseq-count [91] version 0.6. Normalization and differential expression were conducted using DESeq2 [92] version 1.18, using the default parameters and options. Differentially expressed genes in the pair-wise comparisons were identified using Wald’s test. The resulting *p*-values were adjusted for the testing of multiple hypotheses using the Benjamini–Hochberg procedure to control the false discovery rate. The false discovery rate threshold was set to 0.05, and only probe sets exhibiting a minimum 2-fold change in mean relative expression were included in the functional analysis. Overrepresentation of gene ontology and Reactome pathways with hypergeometric testing was determined using ClueGO version 2.5.4, with false discovery rate *p*-value correction [93].

### 4.7. miRNA Data Analysis

Unmapped bam files were converted to fastq files using a bamToFastq script from bedtools version 2.26 [94]. Read mapping to the human genome (hg19), quantification of known miRNAs (according to miRBase release 21), and prediction of novel miRNAs were performed using miRDeep2 version 2.0.0.7 [95]. Differential expression of miRNAs was analyzed using edgeR version 3.20.6 [96], as described previously [87].

### 4.8. Integrated Data Analysis

Transcriptome and miRNA data integration (including PLS-DA and correlation computation) were conducted using DIABLO with mixOmics functions [97] package (mixOmics version 6.8.5). The tuning of the component and the variable number was performed according to the tutorial at http://mixomics.org/mixdiablo/case-study-tcga/.

## 5. Conclusions

To sum up, our study was the first to compare directly and simultaneously mutational and transcriptional profiles of well-differentiated neuroendocrine tumors and ductal adenocarcinomas of the pancreas using massively parallel sequencing. We confirmed the presence of two driver genes, *MEN1* and *DAXX*, mutations in which were found in one-fifth of PNET samples but not in other tumor types. Among the most-mutated genes, *KRAS* and *TP53* were mutated only in cancers, while five other genes (*HNF1A, CREBBP, RNF213, CASC5*, and *APC*) were mutated with similar frequencies in adenocarcinomas and neuroendocrine tumors. However, most other driving mutations were found only in individual tumor samples or in small percentages of tumors. In contrast to the genetic findings, the clearest differences were transcriptomic. While differentially expressed genes may indicate different sites of origin for adenocarcinomas and neuroendocrine tumors, they cannot be linked to significant differences in the aggressiveness of PNETs and PDACs. Finally, our study revealed that both mRNA and miRNA profiles had a high ability to discriminate between tumor samples. In addition, integrative analysis using the novel algorithm DIABLO allowed us to construct a multi-omic biomarker model, based on quantitative data from miRNA and mRNA. Both profiles showed high discriminatory powers, mainly in the second component, where they reached AUC of 1 for all the groups. Unsurprisingly, the miRNA profile was also highly correlated to mRNA expression. Hence, miRNAs can be considered as a new class of pancreatic tumor biomarkers due to their high stability.

## Figures and Tables

**Table 1:** Detailed characteristics of the patients.

Sex	Age	Tumor Location	Tumor Size (cm)	Tumor Type/Grade	Vascular Invasion	Nerve Invasion	Surgery	TNM Stage
M	70	Tail	1.9	PNET G2	+		resection	T4N0M0
F	35	Tail	3.5	PNET G2	+	+	resection	T2N1M0
F	27	Head	3.5	PNET G2	+	+	resection	T2N1M0
F	27	Tail	2.3	PNET G2	+		resection	T2N0M0
F	54	Tail	4.2	PNET G1	+	+	resection	T3N1M1
F	69	Head	4.2	PNET G2	+		resection	T3N1M0
F	44	Tail	3.2	PNET G1	+		resection	T2N0M0
F	24	Head	1.3	PNET G2		+	resection	T1N1M0
F	60	Body	Unknown	PNET G3			laparotomy	Unknown
M	67	Duodenum	3	INET G1			resection	T2N0M0
F	66	Small intestine	2	INET G1			resection	T4N1M0
M	59	Small intestine	2.7	INET G1			resection	T3N0M1
M	38	Small intestine	1.7	INET G1			resection	T1N0M0
M	45	Head	2.8	PDAC G2	+	+	resection	T2N2M0
F	72	Body	Unknown	PDAC G?	+		laparotomy	T4N1M0
F	51	Head	2.5	PDAC G2	+		resection	T2N2M0
F	67	Head	3.3	PDAC G3	+	+	resection	T4N2M0
F	69	Head	3	PDAC G2	+	+	resection	T2N2M0
M	38	Tail	1.7	PDAC G?			resection	T1N0M1
F	70	Head	Unknown	PDAC G?	+		laparotomy	T3N1M1
F	71	Head	Unknown	PDAC G?	+		laparotomy	T4N1M0

PNET—Pancreatic neuroendocrine tumor, PDAC—pancreatic ductal adenocarcinoma, INET—intestinal neuroendocrine tumor.

**Table 2:** Distribution of likely deleterious variants among the three tumor types.

Names	Total	Elements
INETs PDACs PNETs	10	*CASC5 PAX7 ATRX NFKB2 HNF1A APC STK11 TSHR CREBBP RNF213*
PDACs PNETs	11	*ARID2 MTOR COL1A1 MLL2 KAT6B BCL9 BLM FN1 NOTCH2 MSH6 ATM*
INETs PDACs	1	*ERBB4*
INETs PNETs	2	*MLL3 LPHN3*
PDACs	28	*EP400 CYP2D6 KRAS ABL2 BCL2 PTPRT TP53 RET KDM5C TET2 ING4 PER1 SMAD4 BCL6 PALB2 NPM1 NUP98 CDKN2A ERG BAP1 MET GATA1 PRDM1 ATR RNASEL FANCG PLEKHG5 KLF6*
PNETs	34	*FGFR3 AURKB CDH1 FLI1 CKS1B CYP2C19 SMO UBR5 TNFAIP3 SOCS1 NTRK1 TNK2 MITF EPHA3 ARID1A MEN1 PIK3CG MLL VHL MAP2K1 MYD88 SF3B1 RPS6KA2 CSF1R BCR RUNX1T1 PRKDC TFE3 JAK3 AR PMS1 ESR1 DAXX NIN*
INETs	11	*FLT4 GDNF NOTCH1 SMARCA4 ZNF521 ITGB2 EP300 PTCH1 FLT3 BIRC3 TSC2*

PNET—Pancreatic neuroendocrine tumor, PDAC—pancreatic ductal adenocarcinoma, INET—intestinal neuroendocrine tumor.

**Table 3:** The most frequently mutated (i.e., with a frequency of more than 40% samples) genes in the three tumor types.

Names	Total	Elements
INETs PDACs PNETs	2	*CREBBP CASC5*
PDACs PNETs	2	*HNF1A APC*
INETs PDACs	1	*RNF213*
PDACs	2	*KRAS TP53*
PNETs	4	*MLL2 KAT6B JAK3 MLL3*
INETs	8	*FLT4 NFKB2 PAX7 FLT3 ZNF521 NOTCH1 EP300 STK11*

PNET—Pancreatic neuroendocrine tumor, PDAC—pancreatic ductal adenocarcinoma, INET—intestinal neuroendocrine tumor.

**Table 4:** AUC (area under the curve) values for the first three components in the PLS-DA Diablo model.

Group	AUC	*p*-Value	Component	Analysis
PNET	0.9	0.003289	1	mRNA
INET	0.625	0.5023	1	mRNA
PDAC	1	0.000386	1	mRNA
PNET	1	0.000239	2	mRNA
INET	1	0.00729	2	mRNA
PDAC	1	0.000386	2	mRNA
PNET	1	0.000239	3	mRNA
INET	1	0.00729	3	mRNA
PDAC	1	0.000386	3	mRNA
PNET	0.8889	0.004267	1	miRNA
INET	0.6458	0.4338	1	miRNA
PDAC	1	0.000386	1	miRNA
PNET	1	0.000239	2	miRNA
INET	1	0.00729	2	miRNA
PDAC	1	0.000386	2	miRNA
PNET	1	0.000239	3	miRNA
INET	1	0.00729	3	miRNA
PDAC	1	0.000386	3	miRNA

PNET—Pancreatic neuroendocrine tumor, PDAC—pancreatic ductal adenocarcinoma, INET—intestinal neuroendocrine tumor.

## Supplementary Materials

The following are available online at https://www.mdpi.com/1422-0067/21/12/4470/s1.
